# A Case of Recurrent Rudimentary Horn Ectopic Pregnancies Managed by Methotrexate Therapy and Laparoscopic Excision of the Rudimentary Horn

**DOI:** 10.1155/2016/5747524

**Published:** 2016-02-07

**Authors:** Gaby N. Moawad, Elias D. Abi Khalil

**Affiliations:** Department of Obstetrics and Gynecology, George Washington University, Washington, DC, USA

## Abstract

This report presents a case of a 31-year-old woman successfully treated medically for a noncommunicating rudimentary horn ectopic pregnancy who presented with a second, successive rudimentary horn pregnancy. Patient underwent laparoscopic excision of right rudimentary horn and right salpingectomy after failed methotrexate therapy. Given the potential for rupture and recurrence, serious efforts should be made to excise a uterine rudimentary horn.

## 1. Introduction

A unicornuate uterus with or without a rudimentary horn is among the rare forms of uterine anomalies. The incidence of a pregnancy in a rudimentary horn is 1 in 76,000–150,000 pregnancies [1]. We report a case involving consecutive rudimentary horn ectopic pregnancies ultimately managed by operative laparoscopy.

## 2. Case Presentation

**Figure 1:**
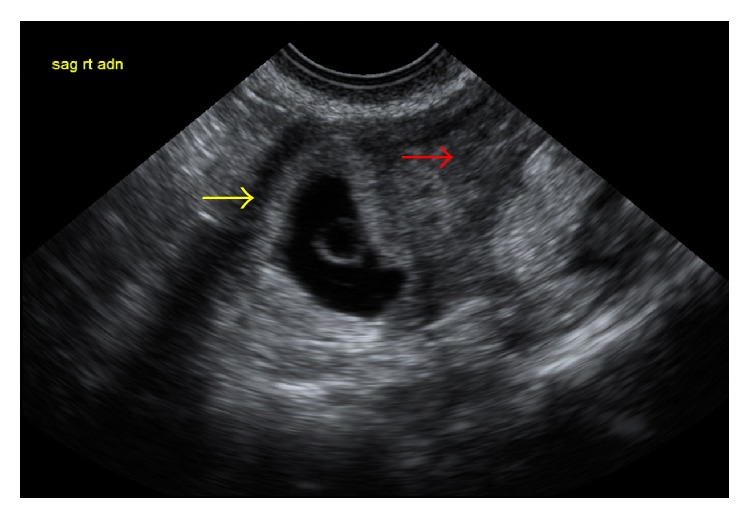
Transvaginal ultrasound. Red arrow: endometrial stripe of 2.4 cm and no visible intrauterine pregnancy. Yellow arrow: right adnexal mass/ectopic pregnancy.

**Figure 2:**
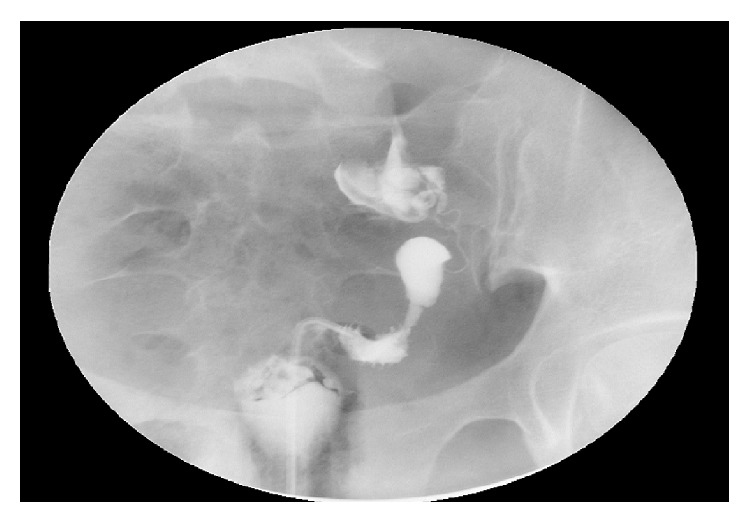
Hysterosalpingogram with unicornuate uterus pushed to the left.

**Figure 3:**
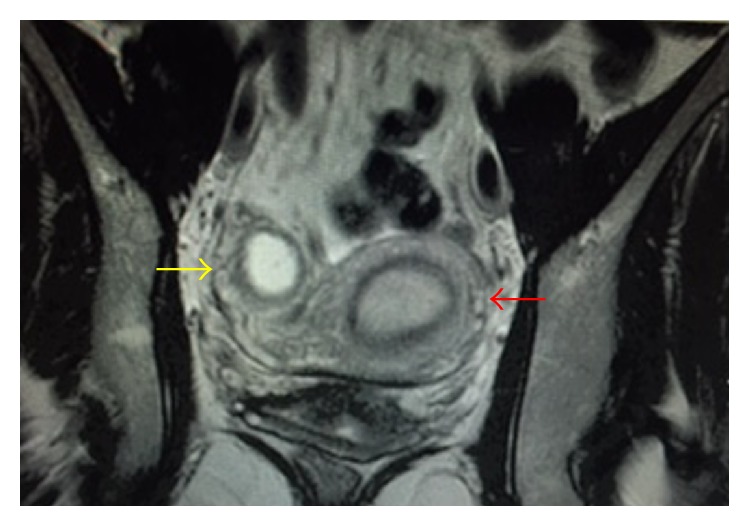
Magnetic Resonance Imaging. Red arrow: left unicornuate uterus. Yellow arrow: noncommunicating right horn with ectopic pregnancy.

A 31-year-old nulliparous presented at five weeks gestation with severe abdominal cramping and nausea of one-day duration. She had no prior history of abdominal surgery or pelvic inflammatory disease. At the time of presentation, her beta-hCG was 45 mIU/mL. A follow-up beta-hCG five days later was 657 mIU/mL. A transvaginal ultrasound performed ten days from her presentation demonstrated an endometrial stripe of 2.4 cm with no intrauterine pregnancy (IUP) seen. An elongated soft tissue structure suggestive of an ectopic pregnancy was noted in the right adnexa (Figure 1). At this time, the patient was successfully treated with methotrexate with a negative two-week B-HCG assay. Due to continuous pelvic pressure, a follow-up ultrasound was ordered a month later and showed a persistent right adnexal mass. A hysterosalpingogram (HSG) revealed a uterine cavity deviated to the left with a patent ipsilateral tube suggestive of a unicornuate uterus (Figure 2). Follow-up Magnetic Resonance Imaging (MRI) confirmed a left unicornuate uterus with a noncommunicating right horn (Figure 3). Intravenous pyelogram revealed normal upper and lower urinary tract system. Two months later, the patient noted a positive home pregnancy test. Her quantitative beta-hCG level was 199 mIU/mL and an ultrasound revealed a 5-week rudimentary horn ectopic pregnancy with a yolk sac. Methotrexate was attempted but failed as her follow-up day 4 and day 7 B-HCG levels were on the rise. An MRI with contrast confirmed the diagnosis of a right rudimentary horn ectopic pregnancy. Patient was counseled for laparoscopic excision of rudimentary horn and right ectopic pregnancy.

**Figure 4:**
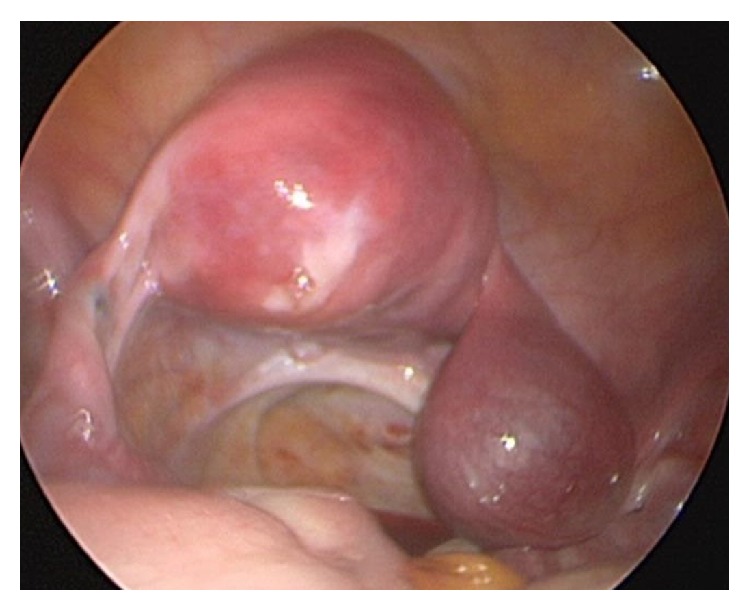
Direct visualization of ectopic pregnancy in right rudimentary horn with left unicornuate uterus.

After placement of a Foley catheter, a V-Care uterine manipulator was inserted in the uterus. A transumbilical Veress needle approach with optical trocar placement was utilized to gain peritoneal entry. Right and left lower quadrant and suprapubic ports were then placed under direct visualization. With patient in Trendelenburg position, the right rudimentary horn ectopic pregnancy and unicornuate uterus were visualized (Figure 4). The right round ligament was transected. The right perirectal space was developed and a right ureterolysis was performed to the ureteric tunnel. The uterine artery was skeletonized and ligated at its hypogastric origin. A window was opened in the posterior leaf of the right broad ligament between the ureter and the right utero-ovarian ligament. The right fallopian tube was released from the ovary and the right uteroovarian ligament was transected. The anterior leaf of the broad ligament was opened to the level of the anterior vaginal fornix allowing the bladder to be reflected well away from the right rudimentary horn. The rudimentary horn was then transected from the main left uterine horn and the small serosal defect repaired intracorporeally with running 2.0 vicryl suture. The resected rudimentary horn and ectopic pregnancy were placed in a 10 mm endobag and removed via the expanded suprapubic port site. After complete hemostasis was noted, the abdomen was desufflated and the port sites closed.

Minimal blood loss was encountered during the case and the patient was discharged home the same day. Pathology confirmed the findings of right rudimentary ectopic pregnancy. A quantitative beta-hCG was negative two weeks after operation.

## 3. Discussion

A unicornuate uterus results from the arrested or defective development of one of the Mullerian ducts [1]. A rudimentary horn exists in seventy-five percent of cases. It results from partial development of one Mullerian duct and incomplete fusion with its contralateral counterpart [2]. In 1979, Buttram Jr. and Gibbons presented a classification system for unicornuate uteri that remain in use today. According to their classification, there are four types: A1a, A1b, A2, and B. Type A is a unicornuate uterus with an associated rudimentary horn on the contralateral side (further subtyped according to the presence or absence of a cavity, A1 and A2, resp.). Type A1 is further subdivided into whether the rudimentary horn cavity is communicating with the uterus or not, A1a and A1b, respectively. Type B is a unicornuate uterus with no contralateral rudimentary horn structure [3, 4].

Knowing that rudimentary horns are not contiguous with the cervix, several theories have been proposed to explain the mechanism of ectopic pregnancies. First theory describes the retrograde entry of sperm into the fallopian tube of the rudimentary horn. This would require sperm to navigate through the unicornuate uterus, the ipsilateral tube, and peritoneal cavity before entry into the rudimentary side. The rate of transperitoneal sperm migration is thought to be as high as 51% in these pregnancies [5]. The second theory proposed by Latto and Norman in 1950 states that microscopic channels may exist between the endometrial and rudimentary horn cavity. These would allow direct passage of sperm from the unicornuate cavity to the rudimentary horn [6].

Fifty percent of unrecognized rudimentary horn ectopic pregnancies rupture before the third trimester [7]. Other complications include preterm labor, fetal malpresentation, and placenta accreta [8]. Early diagnosis is a crucial step in preventing these complications. Combined quantitative hCG and sonographic assessments remain the mainstay for diagnosis. Sonographic criteria for diagnosing rudimentary horn ectopic pregnancies have been described and these included: a pseudopattern of an asymmetrical bicornuate uterus, absent visual continuity of tissue surrounding the gestational sac and the uterine cervix, and the presence of myometrial tissue surrounding the gestational sac [9]. Although not helpful during pregnancy, hysterosalpingography can assist in diagnosing a unicornuate uterus by showing filling of a small, fusiform uterine cavity that tapers at the apex and is seen shifted to one side of the pelvis. It can also diagnose a rudimentary horn of the communicating type. A noncommunicating rudimentary horn cannot be visualized by HSG and these cases are usually diagnosed by MRI [10].

When diagnosed early, medical management with methotrexate has been reported [11]; however, it does not prevent recurrence as demonstrated by the case presented. Surgical management allows for definitive treatment of the ectopic pregnancy and prevents recurrence. Traditionally, rudimentary horn pregnancies have been surgically managed via laparotomy with excision of the rudimentary horn and the ipsilateral fallopian tube [2]. With current advances in laparoscopic technique, it is possible to manage these cases laparoscopically. Given the potential for rupture and recurrence, serious efforts should be made to excise the rudimentary horn. This can be done at the time of ectopic pregnancy diagnosis or few weeks after methotrexate therapy when tissues are less fragile and have fewer tendencies to bleed.
